# Efficacy and safety of immune checkpoint inhibitors in patients with advanced intrahepatic cholangiocarcinoma

**DOI:** 10.3389/fonc.2025.1498887

**Published:** 2025-02-05

**Authors:** Ziting Peng, Jianhui Dong, Shuyao Tang, Jiaxu Shi, Tongdong Shi

**Affiliations:** ^1^ Department of Infectious Diseases, Key Laboratory of Molecular Biology for Infectious Diseases (Ministry of Education), Institute for Viral Hepatitis, the Second Affiliated Hospital, Chongqing Medical University, Chongqing, China; ^2^ First Clinical College of Chongqing Medical University, Chongqing, China

**Keywords:** intrahepatic cholangiocarcinoma, PD-1 antibody, PD-L1 antibody, immune checkpoint inhibitors, immunotherapy

## Abstract

**Background:**

To assess the efficacy and safety of PD-1 and PD-L1 immune checkpoint inhibitors (ICIs) in managing advanced intrahepatic cholangiocarcinoma (ICC).

**Methods:**

A retrospective analysis of treatment data for patients with advanced ICC who received ICIs at the Second Affiliated Hospital of Chongqing Medical University from the inception of the inpatient medical record database until 30 April 2024. The analysis concentrated on the safety and efficacy of the treatment. The primary endpoint was progression-free survival (PFS), while the secondary endpoints included overall survival (OS) and safety. The Kaplan-Meier method was employed to plot survival curves, and differences between groups were assessed using log-rank tests.

**Results:**

96 patients diagnosed with ICC were included, comprising 60 males (62.50%) and 36 females (37.50%). 85 patients exhibited disease progression, 22 patients succumbed, and 38 patients were lost to follow-up finally. Those who initiated immunotherapy promptly following first-line antitumor treatment exhibited a notably prolonged PFS compared to those experiencing tumor progression (5.63 months (*95%CI:* 3.12~8.14) vs 2.50 months (*95%CI:* 1.83~3.17), *P=*0.002). However, no significant disparity in the PFS with immunotherapy in different lines therapy(*P=*0.406) and the OS was observed between the two groups(*P=*0.360). 18 patients (18.75%) experienced treatment-emergent adverse events (AEs), with 3 patients encountering AEs of grade ≥3. All patients returned to normal after symptomatic treatment.

**Conclusions:**

In patients with advanced ICC, the timely initiation of ICIs as adjuvant therapy following first-line antitumor treatment can result in favorable efficacy and a good safety profile.

## Introduction

Intrahepatic cholangiocarcinoma (ICC) is a type of adenocarcinoma that originates from the epithelium of the secondary and upper bile duct branches within the liver. The incidence of ICC is second only to that of hepatocellular carcinoma (HCC) (1). It is notable that ICC has a greater tendency to invade and metastasize compared to HCC, leading to significantly shorter OS for ICC patients (2). Surgical resection remains the preferred treatment for ICC patients. However, because early symptoms are often non-specific, many patients do not seek medical attention in time for surgery at the initial diagnosis. Furthermore, ICC is highly malignant, with low rates of surgical resection and a recurrence rate of 60-70% within five years after surgery (3). In recent years, the incidence and mortality rates of ICC have increased globally, with particularly high rates observed in Asian populations compared to those in Europe and North America (4, 5).

The effectiveness of the chemotherapy combination of Gemcitabine and Cisplatin (GP) remains limited (6). In recent years, ICIs have demonstrated promising results in the treatment of various malignancies, including colorectal cancer, non-small cell lung cancer, and malignant melanoma. By inhibiting the protein expression of immunosuppressive checkpoints, ICIs reduce immunosuppression and enhance T-cell activity, ultimately enabling the destruction of cancer cells and the production of an anti-tumor response (7, 8). Among these, inhibitors targeting programmed cell death protein 1 (PD-1) and programmed cell death ligand 1 (PD-L1) are the most extensively utilized. PD-1 is expressed in activated T cells, while PD-L1 is expressed on the surfaces of various immune system cells (9). Moreover, high PD-L1 expression is closely related to low histological differentiation, advanced stage, and poor prognosis of the tumor (10). Therefore, PD-L1 expression can be used as an indicator to assess the malignancy and prognosis of cholangiocarcinoma.

Many patients with advanced ICC have no indication for surgical or biological resection, and the prognosis for these patients is poor. It is our objective to improve their survival rate and extend their lifespans as much as possible. We aim to achieve this through the use of ICIs. However, there is a lack of clinical trial data on the efficacy of these agents in ICC, as well as on the optimal timing for initiating immunotherapy. Whether early ICIs administration can improve outcomes for these patients? Therefore, this study aims to evaluate the efficacy and safety of PD-1 and PD-L1 antibodies in the treatment of advanced ICC.

## Materials and methods

**Figure 1:**
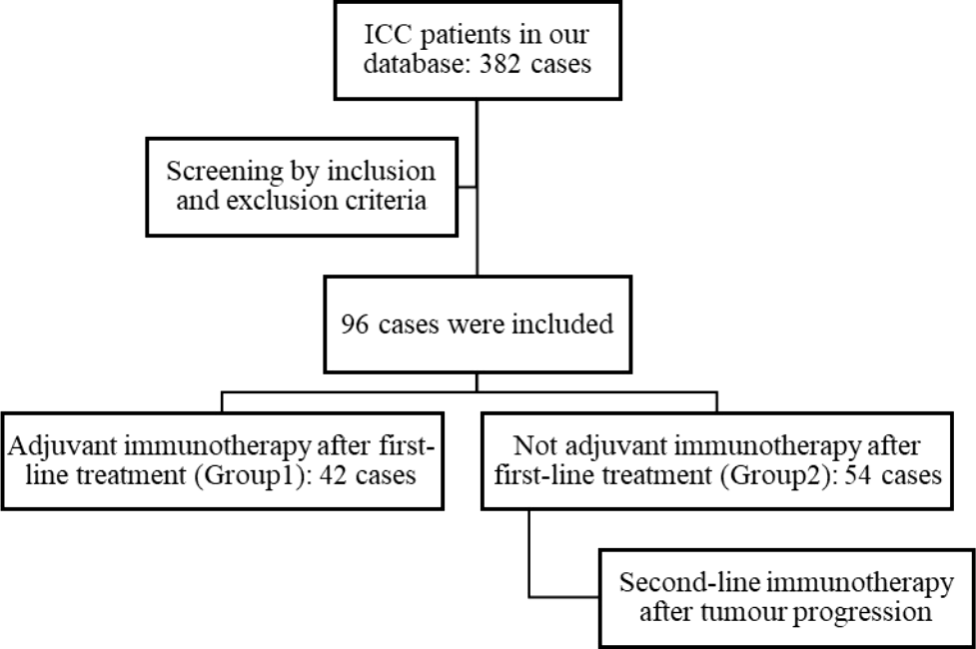
Experimental design and grouping flowchart.

1. Study subjects: Review and collect electronic data of patients at the Second Affiliated Hospital of Chongqing Medical University who have received PD-1 and PD-L1 antibody drug therapy for advanced ICC since their admission to the present date of 30 April 2024. Inclusion criteria: ①age≥18 years; ②pathological examination confirmed the diagnosis of ICC; ③Eastern Cooperative Oncology Group (ECOG) score ≤ 2 points; ④stage II to IV ICC patients who had undergone first-line treatment (tumor resection, local treatments such as transcatheter arterial chemoembolization (TACE), radiofrequency ablation (RA), high-intensity focused ultrasound (HIFU) therapy, and particle implantation, radiation therapy, chemotherapy, symptomatic treatment), and there is no evidence to suggest that radical surgical excision is a viable option for surgical or functional resection at present; ⑤Child-Pugh classification standard is A-B; ⑥never used ICIs before. Exclusion criteria: ①combined with other malignant tumors or serious illnesses that may affect a patient’s life, such as cardiopulmonary, liver and kidney failure, serious progressive infection; ②previously received anti-tumor immunotherapy; ③those not adhering to a regular medication regimen; ④those with contraindications to the use of ICIs.
2. Treatment procedure: A total of 96 ICC patients were enrolled in the study. Of these, 42 patients received immunotherapy as adjuvant treatment following first-line surgery, local therapy, chemoradiotherapy, etc. (Group1), while 54 patients were treated after ICC progression (Group2). (**Figure 1**) 48 patients received ICIs as monotherapy, 16 patients received ICIs in combination with Lenvatinib, 30 patients were treated with ICIs alongside chemotherapy, and 2 patients received a regimen combining ICIs, Lenvatinib, and chemotherapy. The PD-1 inhibitors included Sintilimab (200mg/dose), Camrelizumab (200mg/dose), Tislelizumab (200mg/dose), and Toripalimab (200mg/dose). The PD-L1 inhibitors included Durvalumab (1000mg/dose). The drugs are administered intravenously once every three weeks. Patients with hepatitis B virus (HBV) infection receive standard antiviral treatment concurrently with immunotherapy.
3. Observation Endpoints: The primary endpoints of this study are progression-free survival (PFS) and overall survival (OS). PFS is defined as the duration from the initiation of immunotherapy to the documented date of disease progression. OS is defined as the time from the initiation of immunotherapy to the date of death from any cause or the date of the last follow-up. Throughout the treatment and follow-up period, the safety profile is assessed by evaluating adverse events (AEs) in accordance with the Common Terminology Criteria for Adverse Events (CTCAE) version 5.0.
4. Data Collection: The clinical information, laboratory test results, and imaging data for the enrolled patients are gathered from the hospital’s electronic medical record system. This includes the patient’s name, gender, age, ECOG performance status, liver function (The Child-Pugh classification standard), TNM stage, pathological biopsy information, lesion characteristics (tumor diameter, intrahepatic and extrahepatic metastasis, lymph node metastasis, vascular invasion), HBV infection, and medication information.
5. Follow-up: Patients received immunotherapy underwent a complete blood count, liver and kidney function tests every 3 weeks, and abdominal enhanced CT or MRI every 3 months. For patients who were not undergo regular imaging, the observation indicators were the significant increase of tumor markers CA199 and CEA indicating disease progression. Follow-up methods included re-visits, medical record reviews, and telephone follow-ups. The most recent follow-up was conducted on 30 April 2024. For patients with disease progression, the endpoint event is defined as the time of tumor progression, while for patients without disease progression, the endpoint event is defined as the end of the study. Patients lost to follow-up are considered to have the endpoint event at the time of their loss to follow-up.
6. Statistical analysis: The SPSS 25.0 software was employed for the processing and analysis of the data. The baseline data of the two groups of patients were compared using the independent samples t-test and the chi-squared test, with a significance of P < 0.05. The Kaplan-Meier method was employed to perform survival analysis and generate survival curves. The statistical significance of differences between groups was evaluated using the log-rank test, with a significance level of P < 0.05.

## Results

**Figure 2:**
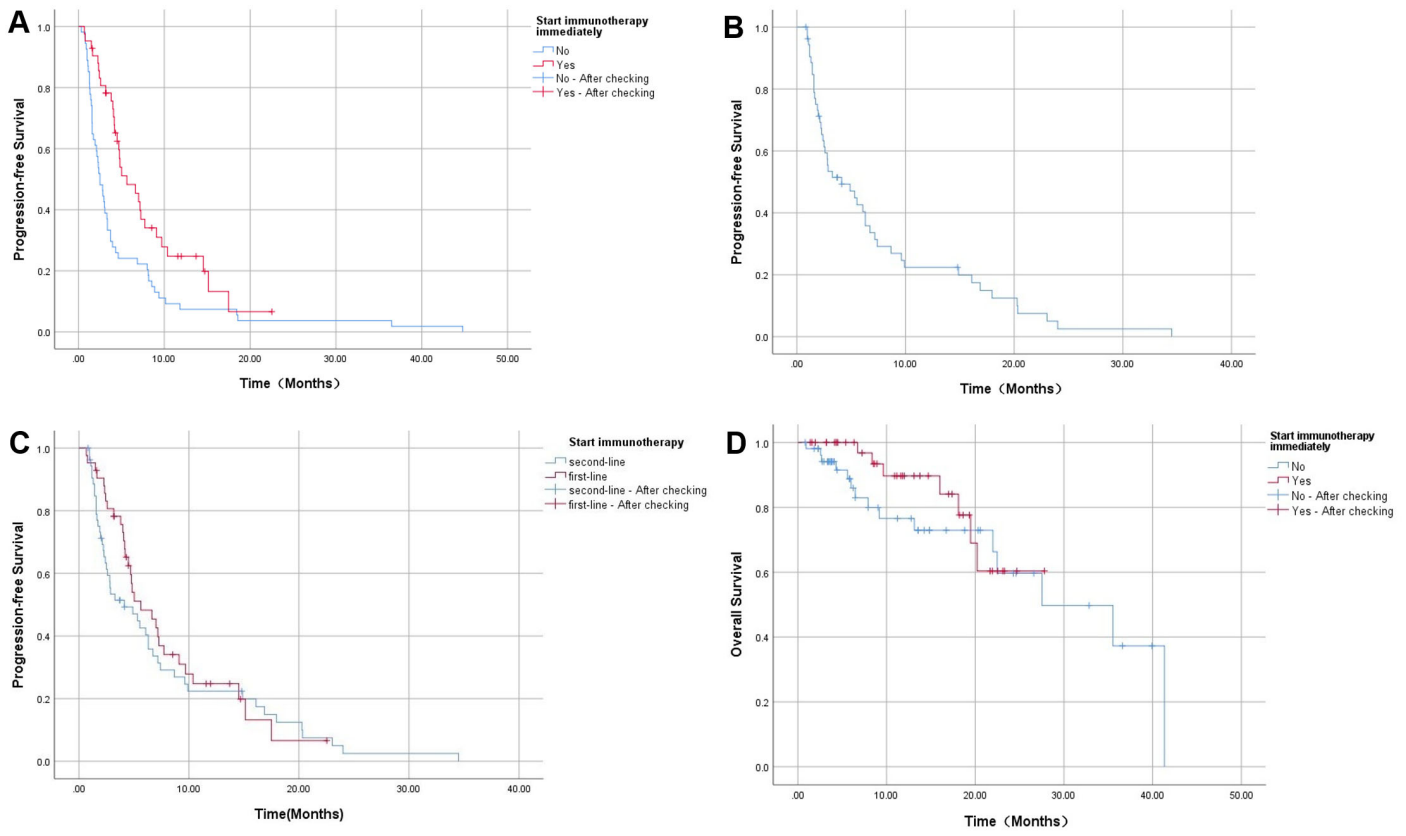
**(A)** The comparison of PFS between the immunotherapy adjunct group and the non-adjunct therapy group; **(B)** PFS of patients receiving immunotherapy after tumor progression; **(C)** The comparison of PFS with immunotherapy in different lines treatment; **(D)** The comparison of OS between the immunotherapy adjunct group and the non-adjunct therapy group.

1. General information: This study included a total of 96 patients. The baseline characteristics of the patients are shown in **Table 1**. The group consisted of 60 males and 36 females. Before using ICIs, 48 patients undergone surgical resection previously; 37 patients received local anti-tumor treatment, including: TACE, RA, HIFU therapy, and particle implantation; 6 patients received radiotherapy; 35 patients received chemotherapy; and 9 patients received symptomatic treatment only. 42 patients were treated with immunotherapy as adjuvant therapy following first-line treatment, while 54 patients were treated following tumor progression. During immunotherapy, 48 patients received single-agent immunotherapy, 16 received combination immunotherapy with Lenvatinib, 30 received combination immunotherapy with chemotherapy, and 2 patients received a triple combination of chemotherapy, immunotherapy and Lenvatinib. Specific medication details are as follows: Sintilimab in 27 cases, Camrelizumab in 27 cases, Toripalimab in 19 cases, Tislelizumab in 18 cases, and Durvalumab in 5 cases.
2. Clinical efficacy: As of 30 April 2024, 85 (88.54%) of the 96 patients experienced disease progression. Among the patients, all 54 patients in Group2 experienced disease progression; Of the 42 patients in Group1, 31 progressed, 7 did not progress, and 4 were lost to follow-up. The median PFS=5.63 months (95%CI: 3.12~8.14) in patients who were initiated immunotherapy immediately after first-line treatment was significantly longer than the PFS=2.5 months (95%CI: 1.83~3.17) in patients who were not (P=0.002) (**Figure 2A**). Among the 54 patients in Group2, the median PFS following immunotherapy was 4.13 months (95%CI: 0.88-7.38) (**Figure 2B**). A comparison of the median PFS of two groups of patients initiated immunotherapy in first-line treatment (5.63 months, 95%CI: 3.12~8.14) and those initiated immunotherapy in second-line treatment (4.13 months, 95%CI: 0.88~7.38) revealed no significant difference between the two groups (P=0.406) (**Figure 2C**). Patients who initiated immunotherapy as adjuvant therapy had too few deaths to estimate OS. The median OS of patients who were treated after disease progression was 27.53 months (95%CI: 13.10~41.96), and the difference in OS between the two groups was not statistically significant (P=0.360) (**Figure 2D**). Of those 96 patients, 22 died (22.92%), 36 survived, and 38 were lost to follow-up. The causes of death included severe infection or septic shock caused by tumor progression (7 cases), gastrointestinal bleeding (4 cases), liver failure (1 case), multiple systemic metastases (5 cases), cancer cachexia (3 cases), and unknown cause (2cases).
3. Adverse events (AEs) and safety: Among the 96 patients, 18 cases (18.75%) experienced adverse events of varying degrees, with 3 cases (3.13%) experiencing AE grade≥3. Among the cases, 9 cases (9.38%) exhibited mild liver function damage, which was primarily manifested as increased levels of AST or ALT; 3 cases (3.13%) developed immune-related rash, presenting as skin itching. Thyroid dysfunction occurred in 2 cases (2.08%), mainly manifested as hypothyroidism. Hyperthermia occurred in 1 case (1.04%), considered to be associated with tumor progression and abdominal infection. 1 case (1.04%) exhibited immune-related pneumonia; 1 case (1.04%) exhibited newly developed hepatic hemangioma; 1 case (1.04%) exhibited thrombocytopenia, considered to be related to the side effects of chemotherapy drugs. All the above AEs recovered without sequelae after symptomatic treatment.

**Table 1:** Patient baseline data.

Characteristics	Group1 (42)	Group2 (54)	*P*
Male/Female【case (%)】	27 (64.3)/15 (35.7)	33 (61.1)/21 (38.9)	0.750
age (years)	56 ± 10.529	58 ± 9.793	0.586
ECOG performance status【case (%)】			0.941
0	12 (28.6)	17 (31.5)	
1	24 (57.1)	29 (53.7)	
2	6 (14.3)	8 (14.8)	
Child-Pugh standard【case (%)】			0.384
A (5-6)	32 (76.2)	45 (83.3)	
B (7-9)	10 (23.8)	9 (16.7)	
HBsAg(+)【case (%)】	10 (23.8)	17 (31.5)	0.407
Liver cirrhosis【case (%)】	4 (9.5)	6 (11.1)	0.801
Pathological differentiation degree 【case (%)】			0.864
poorly differentiated	9 (21.4)	14 (25.9)	
moderately to poorly differentiated	8 (19.0)	11 (20.3)	
moderately differentiated	18 (42.8)	22 (40.7)	
moderately to well differentiated	2 (4.7)	3 (5.5)	
well differentiated	3 (7.1)	1 (1.8)	
unclear	2 (4.7)	3 (5.5)	
number【case (%)】			0.595
single	25 (59.5)	35 (64.8)	
multiple	17 (40.4)	19 (35.1)	
tumor characteristics【case (%)】			
vascular invasion	18 (42.8)	22 (40.7)	0.835
lymph node metastasis	30 (71.4)	31 (57.4)	0.157
intrahepatic and extrahepatic metastasis	21 (50.0)	35 (64.8)	0.203
maximum diameter【case (%)】			0.824
≤2cm	3 (7.1)	5 (9.3)	
2-5cm	17 (40.5)	24 (44.4)	
5-10cm	20 (47.6)	24 (44.4)	
>10cm	2 (4.8)	1 (1.9)	
TNM stage【case (%)】			0.887
II	5 (11.9)	6 (11.1)	
IIIA	4 (9.5)	3 (5.5)	
IIIB	17 (40.4)	22 (40.7)	
IV	16 (38.0)	23 (42.5)	

(gender, age, ECOG performance status, The Child-Pugh classification standard, TNM stage, pathological biopsy information, tumor diameter, intrahepatic and extrahepatic metastasis, lymph node metastasis, vascular invasion, HBV infection).

## Discussion

In recent years, immunotherapeutic drugs have brought about significant advances in cancer therapy, with 10% to 20% of ICC patients who were resistant to chemotherapy achieving remission following immunotherapy (11). Current immune-related therapies include ICIs targeting PD-1, PD-L1, and cytotoxic T-lymphocyte-associated protein 4 (CTLA-4). Vaccines, adoptive cell therapy and non-specific immunomodulators are also available. Among these, PD-1 and PD-L1 antibody drugs are the most widely used. However, there are differences between them in terms of mechanism of action, clinical efficacy, adverse reactions, cost, and other factors. In our research, most patients were treated with domestic PD-1 inhibitors, likely influenced by the availability of healthcare coverage for such medications and the financial circumstances of the patients.

Currently, there are many limitations in the study of immunotherapy in cholangiocarcinoma, and comprehensive studies on the optimal timing, indications and efficacy of different drugs are still lacking. Both KEYNOTE-028 and KEYNOTE-158 studies suggest that ICIs have a significant impact on the treatment of advanced cholangiocarcinoma (12). However, most patients are often treated with multiple regimens in the real world, and the efficacy of single-agent immunotherapy in advanced cases remains limited. This study includes various treatment regimens, such as single-agent immunotherapy, doublet therapy, and triplet therapy. A retrospective study from China showed that PD-1 inhibitor combined with Lenvatinib prolonged survival in those with advanced cholangiocarcinoma who had not responded to chemotherapy (13). The results of the follow-up TOPAZ-1 trial indicate that patients with advanced cholangiocarcinoma exhibited a notable improvement in OS, PFS, and ORR when treated with a combination of Durvalumab and the GP regimen and the drug’s safety profile was within acceptable range (14). This is the world’s first successful Phase III clinical trial to combine chemotherapy with immunotherapy as a first-line treatment for cholangiocarcinoma. Currently, Durvalumab combined GP regimen has been adopted by definitive guidelines and is recommended as a first-line treatment option (15, 16).

In 2023, Zhou Jian’s team reported a triple combination regimen of chemotherapy, targeted therapy, and immunotherapy with the monoclonal antibody Toripalimab combined with Lenvatinib and the chemotherapy regimen GEMOX (gemcitabine + oxaliplatin) for the treatment of advanced ICC. The results demonstrated an 80% ORR, with median OS and PFS of 22.5 months and 10.2 months, respectively. These findings represent a significant improvement in efficacy compared to previous monotherapy or combination therapy regimens, with manageable AEs occurring in over half of the patients. This suggests that the treatment of advanced ICC can be gradually extended to chemotherapy, immunotherapy and targeted triple drug (17).

This study included 96 patients with advanced ICC, of whom 85 experienced disease progression. The median PFS of patients who received PD-1 or PD-L1 inhibitors as adjuvant therapy after prior first-line treatment was longer than that of those who did not receive active immunotherapy {5.63 months (95%CI: 3.12~8.14) VS 2.5 months (95%CI: 1.83~3.17)}(P=0.002). This suggests that once diagnosed with ICC, the proactive use of ICIs at an early stage will significantly enhance patient prognosis in comparison to initiating immunotherapy after tumor progression. This indicates that anti-tumor immunotherapy should be introduced as early as possible. For the 54 patients treated after tumor progression, the mPFS after immunotherapy was 4.13 months (95%CI: 0.88~7.38), suggesting that ICIs as a post-line treatment can also appropriately prolong PFS. However, the lack of controlled trials comparing the two groups in second-line treatment introduces some potential bias into the conclusions. Due to the aggressive nature of ICC and its rapid progression, the PFS with immunotherapy in different lines therapy and the final OS between the two groups were not significantly different. In terms of safety, 18 (18.75%) of the 96 patients who experienced AEs recovered following conservative treatment, and there were no deaths related to the treatment. In conclusion, ICIs were well tolerated in ICC therapy and had controlled toxic reactions.

This study still has several limitations. Firstly, it is challenging to rigorously control a single variable in real-world. Some patients received targeted therapies or chemotherapy, which introduces a degree of bias into the efficacy outcomes. Secondly, the study employed a diverse range of drugs, and the sample size was limited, which precluded further stratification of drug types. Most of the advanced ICC patients were in critical condition at the time of treatment, with rapid tumor progression. Additionally, some patients requested to cease treatment, resulting in a significant number of lost follow-ups. The study on PFS of patients undergoing immunotherapy with ICIs after relapse and metastasis is a single-center study lacking a control trial. PD-1 and PD-L1 inhibitors have a potential to significantly extend PFS and appear to have a favorable safety profile for treating advanced ICC patients. Subsequent randomized, large-scale, and prospective trials are essential to optimize the use of immunotherapy in these ICC patients.

## Data Availability

The original contributions presented in the study are included in the article/supplementary material. Further inquiries can be directed to the corresponding author.
